# Low-pass spectral analysis of time-resolved serial femtosecond crystallography data

**DOI:** 10.1063/4.0000178

**Published:** 2023-05-26

**Authors:** Cecilia M. Casadei, Ahmad Hosseinizadeh, Spencer Bliven, Tobias Weinert, Jörg Standfuss, Russell Fung, Gebhard F. X. Schertler, Robin Santra

**Affiliations:** 1 Laboratory of Biomolecular Research, Biology and Chemistry Division, Paul Scherrer Institute, Villigen PSI, Switzerland; 2 Institute of Molecular Biology and Biophysics, Department of Biology, ETH Zürich, Zürich, Switzerland; 3 Department of Physics, University of Wisconsin-Milwaukee, Milwaukee, Wisconsin 53211, USA; 4 Science IT Infrastructure and Services, Division Scientific Computing, Theory and Data, Paul Scherrer Institute, Villigen PSI, Switzerland; 5 Center for Free-Electron Laser Science CFEL, Deutsches Elektronen-Synchrotron DESY, 22607 Hamburg, Germany; 6 Department of Physics, Universität Hamburg, 22607 Hamburg, Germany

## Abstract

Low-pass spectral analysis (LPSA) is a recently developed dynamics retrieval algorithm showing excellent retrieval properties when applied to model data affected by extreme incompleteness and stochastic weighting. In this work, we apply LPSA to an experimental time-resolved serial femtosecond crystallography (TR-SFX) dataset from the membrane protein bacteriorhodopsin (bR) and analyze its parametric sensitivity. While most dynamical modes are contaminated by nonphysical high-frequency features, we identify two dominant modes, which are little affected by spurious frequencies. The dynamics retrieved using these modes shows an isomerization signal compatible with previous findings. We employ synthetic data with increasing timing uncertainty, increasing incompleteness level, pixel-dependent incompleteness, and photon counting errors to investigate the root cause of the high-frequency contamination of our TR-SFX modes. By testing a range of methods, we show that timing errors comparable to the dynamical periods to be retrieved produce a smearing of dynamical features, hampering dynamics retrieval, but with no introduction of spurious components in the solution, when convergence criteria are met. Using model data, we are able to attribute the high-frequency contamination of low-order dynamical modes to the high levels of noise present in the data. Finally, we propose a method to handle missing observations that produces a substantial dynamics retrieval improvement from synthetic data with a significant static component. Reprocessing of the bR TR-SFX data using the improved method yields dynamical movies with strong isomerization signals compatible with previous findings.

## I. INTRODUCTION

Time-resolved serial femtosecond crystallography (TR-SFX) is emerging as a prominent technique to investigate the structural dynamics of light-sensitive proteins, at the atomic level with subpicosecond time resolution.^1–4^ In a typical experiment, protein microcrystals embedded in a viscous medium are brought into the interaction region in a continuous fashion. Crystals are photoexcited by a pump laser pulse, and, after a certain time delay, the structure is probed by an x-ray pulse from a free-electron laser (FEL). Since this interaction is destructive, no more than one diffraction pattern can be obtained from one crystal. A dataset consists of tens of thousands of diffraction patterns from individual crystals in random orientations. Only a small fraction of the reciprocal lattice points in the resolution range of interest is measured in an individual diffraction pattern (frame), giving rise to significant data incompleteness. In addition, other stochastic effects such as partiality (that is, stochastic weighting of intensities) and photon counting errors affect the data.

Stochasticity is commonly dealt with by binning-and-merging.^5^ In this approach, the pump-probe delay range is divided into time bins. Equivalent reflections from all frames within a time bin are averaged. This allows to mitigate the stochastic errors and, at the same time, obtain a complete set of intensities per bin, which can be subsequently used, together with intensities from the protein resting state, to calculate difference-electron density maps, which highlight the light-induced structural changes. Numerous studies have been published to date where the ultrafast dynamics of light-sensitive proteins could be accessed by binning-and-merging TR-SFX data (see, for example, Refs. 6–12). Yet, this approach can only provide limited dynamical information. Effective mitigation of stochasticity by averaging typically requires a large number of frames (of the order of tens of thousands, i.e., a significant fraction of the entire dataset), thus resulting in broad time bins. This can imply a loss of timing information, in favorable conditions with relatively small timing uncertainty and short pump and probe pulse durations.

In this work, we explore the application of a recently proposed dynamics retrieval method, the low-pass spectral analysis (LPSA), to the processing of TR-SFX data. LPSA is presented in detail in Ref. 13, where it is shown that excellent dynamics retrieval can be achieved from synthetic data affected by extreme data incompleteness and partiality. The quality of the reconstructed dynamics is found to be superior to that from binning-and-merging. Compared to other sophisticated dynamics retrieval techniques, the singular spectrum analysis (SSA),^14^ and the nonlinear Laplacian spectral analysis (NLSA),^15,16^ a similar reconstruction quality is achieved by LPSA, but with significant computational advantages.

The purpose of the present work is to present and discuss the LPSA of an experimental TR-SFX dataset. After adaptation of the formalism from Ref. 13, to explicitly account for nonhomogeneous time labels (Sec. II), we present the LPSA of the TR-SFX dataset published in Ref. 10, collected from the membrane protein bacteriorhodopsin (bR) (Sec. III). In Sec. IV, we use a synthetic dataset to rationalize our findings from the experimental data. Simulated data offer the opportunity to isolate individual pathologies that are known to affect TR-SFX data and investigate their impact on the analysis. In Sec. IV A, we clarify how increasing timing uncertainty (pump-probe jitter) affects the accuracy of dynamics retrieval. The outcomes of increasing levels of homogeneous data incompleteness and pixel-dependent incompleteness are analyzed in Secs. IV B and IV C, respectively. In Sec. IV D, we account for the effect of photon counting errors by introducing Gaussian noise into the dataset. In Sec. IV E, we propose an alternative procedure to handle missing observations, which is of particular interest when data contain a relevant static component, and apply it to our bR TR-SFX dataset in Sec. V. TR-SFX LPSA results are discussed in Sec. VI, based on the insights gained from the synthetic data.

## II. FORMALISM

While the time evolution of the system gives rise to an intrinsically one-dimensional data manifold, various sources of stochasticity impact the diffraction experiment, making the data space trajectory of the dynamical system unrecognizable. Such effects can be mitigated by using a combination of data concatenation and data filtering by subspace projection in supervector space. This can be achieved, for instance, by using a set of data-driven basis functions, as is done in NLSA. An alternative approach is LPSA, where a set of orthonormal trigonometric functions is used as subspace basis.^13^ In this section, we summarize the LPSA formulation from Ref. 13 and extend it to general, not necessarily uniformly distributed single-frame time labels.

We consider *m* reciprocal lattice points in the resolution range of interest, and denote with *x_ij_* the diffraction intensity related to the lattice point *i* at time point *t_j_*. The *m*-tuple $x_{j}={\left(x_{1j},\ldots ,x_{mj}\right)}^{T}$, thus, represents the diffraction pattern at time point *t_j_*. As a consequence of timing uncertainty, a measured *t_j_* from the experiment generally differs from the time point ${\tilde{t}}_{j}$ that the vector $x_{j}$ truly corresponds to. Since the ${\tilde{t}}_{j}$ is unknown, the ordering of the measured time points *t_j_* is used to order the vectors, i.e., the index *j* of $x_{j}$ reflects the ordering,

$$t_{1}\leq t_{2}\leq \cdots \leq t_{j}\leq \cdots \leq t_{S},$$

for a total of *S* sampled time points.

### A. *q*-fold concatenation

Through *q*-fold concatenation, one obtains from data vectors $x_{j}\in {\mathbb{R}}^{m}$ (super-)vectors $X_{\nu }\in {\mathbb{R}}^{mq}$. Hence, we form

$$X_{1}=\left(\begin{array}{c} x_{q}\\ \vdots \\ x_{1} \end{array}\right),\ldots ,X_{\nu }=\left(\begin{array}{c} x_{\nu +q-1}\\ \vdots \\ x_{\nu } \end{array}\right),\ldots ,X_{S-q+1}=\left(\begin{array}{c} x_{S}\\ \vdots \\ x_{S-q+1} \end{array}\right).$$ (1)

The mean time point associated with $X_{\nu }$ is

$${\bar{t}}_{\nu }=\frac{1}{q}\sum_{j=\nu }^{\nu +q-1}t_{j}.$$ (2)

For a sufficiently large concatenation parameter *q*, the statistical uncertainty of the ${\bar{t}}_{\nu }$ is small in comparison to the statistical uncertainty of the nominal time points *t_j_*. In this sense, the time labels of the supervectors $X_{\nu }$ can be made much more accurate than the time labels of the data vectors $x_{j}$, assuming that the timing uncertainties are primarily statistical. Note, however, that through the construction described by Eq. (1), the uncertainty of the *t_j_* remains reflected in the ordering of the data vectors appearing in each supervector.

### B. Low-pass filtering

Because data incompleteness, partiality, timing uncertainty, and noise compromise the components of the $X_{\nu }$, the one-dimensional manifold describing the dynamical evolution of the system is not readily recognizable. To alleviate these issues, the low-pass projector $\Phi \Phi^{T}\in {\mathbb{R}}^{s\times s}$, with s=S−q+1, was introduced in Ref. 13. By explicitly considering the set of mean measured times $\left\{{\bar{t}}_{\nu }\right\}$, the projector is now built using

$$\psi_{\nu ,2j}=\text{cos}\;(j\omega {\bar{t}}_{\nu }),$$ (3)

$$\psi_{\nu ,2j+1}=\text{sin}\;(j\omega {\bar{t}}_{\nu }),$$ (4)

$$\omega =\frac{2\pi }{{\bar{t}}_{s}-{\bar{t}}_{1}},$$ (5)

with ν=1,…,s; $j=1,\ldots ,j_{\text{max}}$ and $\psi_{1}$ is a constant vector. Orthonormalization of the vectors $\psi_{1},\ldots ,\psi_{2j_{\text{max}}+1}$ yields the columns of Φ. A reduced data representation, where only frequency components below and including the cutoff value $j_{\text{max}}\omega$ are retained, is given by

A=XΦ, (6)

with $A\in {\mathbb{R}}^{mq\times k}$ and $k=2j_{\text{max}}+1$.

### C. Modal decomposition

The reduced data representation is singular-value decomposed

$$A=U\Sigma V^{T},$$ (7)

where $U=(u_{1},\ldots ,u_{r})$, with $u_{i}$ columns of $U\in {\mathbb{R}}^{mq\times r}$; $\Sigma \in {\mathbb{R}}^{r\times r}$, with elements $\Sigma_{ij}=\sigma_{i}\delta_{ij}$; $V=(v_{1},\ldots ,v_{r})$ with $v_{i}$ columns of $V\in {\mathbb{R}}^{k\times r}$; and r≤k is the rank of ***A***. In this framework, we compute the matrix

$$\tilde{X}=\sum_{i=1}^{\tilde{r}}\sigma_{i}u_{i}w_{i}^{T}\in {\mathbb{R}}^{mq\times s},$$ (8)

whose columns ${\tilde{X}}_{\nu }$ capture the points $X({\bar{t}}_{\nu })$ on the manifold more faithfully than the columns of the original ***X*** do. Here, $\tilde{r}$ is the number of LPSA modes retained, and the chronograms $w_{i}=\Phi v_{i}\in {\mathbb{R}}^{s}$ are orthonormal basis vectors for the subspace of relevant functions of time, sampled at the time points ${\bar{t}}_{\nu }$.

### D. Trajectory reconstruction in data space

According to Eq. (1), for S≫q, most data vectors $x_{j}$ appear in the original supervector matrix X *q* times. However, in the matrix $\tilde{X}$ obtained in Eq. (8), this property is not preserved. Therefore, there is no unique way to recover from $\tilde{X}$ the trajectory in data space. Consider the *μ*th *m*-component block in the *ν*th column of $\tilde{X}$

$${\tilde{X}}_{\nu }^{\left(\mu \right)}=\sum_{i=1}^{\tilde{r}}\sigma_{i}u_{i}^{\left(\mu \right)}w_{\nu ,i}\in {\mathbb{R}}^{m}.$$ (9)

Let us assume that *q* is an odd number. Then, Eq. (1) would suggest that the vector

$${\tilde{x}}_{\nu }=\frac{1}{2p+1}\sum_{\mu =-p}^{p}{\tilde{X}}_{\nu +\mu }^{\left([q-1]/2+1+\mu \right)}$$ (10)

is the same, irrespective of whether one chooses *p* = 0, or *p* = 1,…, or p=(q−1)/2. Hence, each of these choices gives a permissible reconstruction of the dynamics in data space. For a *p* chosen, we associate with ${\tilde{x}}_{\nu }$ the time point

$${\tilde{\bar{t}}}_{\nu }=\frac{1}{2p+1}\sum_{\mu =-p}^{p}{\bar{t}}_{\left(\nu +[q-1]/2+\mu \right)}.$$ (11)

The choice p=(q−1)/2 corresponds to the standard reconstruction, also present in SSA^14^ and NLSA.^15^

## III. DYNAMICS RETRIEVAL FROM A TR-SFX DATASET

In this section, we present the LPSA of a TR-SFX dataset from bR in the first picosecond after photoactivation. The interested reader is referred to Ref. 10 for a detailed description of sample preparation, experimental conditions, binning-and-merging procedure, and results.

### A. Data collection

Data were collected at the Linac Coherent Light Source of the SLAC National Accelerator Laboratory from bR microcrystals in the lipidic cubic phase.^17^ The sample was delivered to the interaction region using a high-viscosity injector.^18^ Crystals were photoexcited using a 529 nm pump laser with a pulse duration of 100 fs. The structure was probed using an XFEL pulse with a duration of 50 fs. For each single-shot pattern recorded, in the following referred to as a frame, the pump-probe delay *t_j_* was measured with a timing-tool.^19^ A dataset from the protein in the resting state was also collected, with no photoactivation of the sample.

### B. Data reduction

Data extraction from the diffraction frames is carried out in *CrystFEL.*^5^ This includes data indexing, with prior detector distance optimization for individual experimental runs, intensity integration, and solution of indexing ambiguity in space group P6_3_.

We use customized code to account for the symmetry of the Laue group and map the diffraction intensities from individual frames to the set of *m* unique reciprocal space triplets (*h*, *k*, *l*). We count *m* = 22 727 unique reflections in the resolution range between 20 and 1.8 Å. Of these, only approximately 2% are measured in an individual frame. Following Refs. 16 and 13, we assign a value of zero to missing observations. That is, we map data incompleteness to matrix sparsity.

Frame-dependent scale factors are calculated in *CrystFEL*^5^ and applied to reflections from individual frames using customized code. A multiplicative frame-dependent constant is employed to account for variations in crystal size and beam flux, and a Debye–Waller factor is used to correct for varying levels of crystal disorder, which result in different intensity decay rates with increasing resolution.

**FIG. 1.**
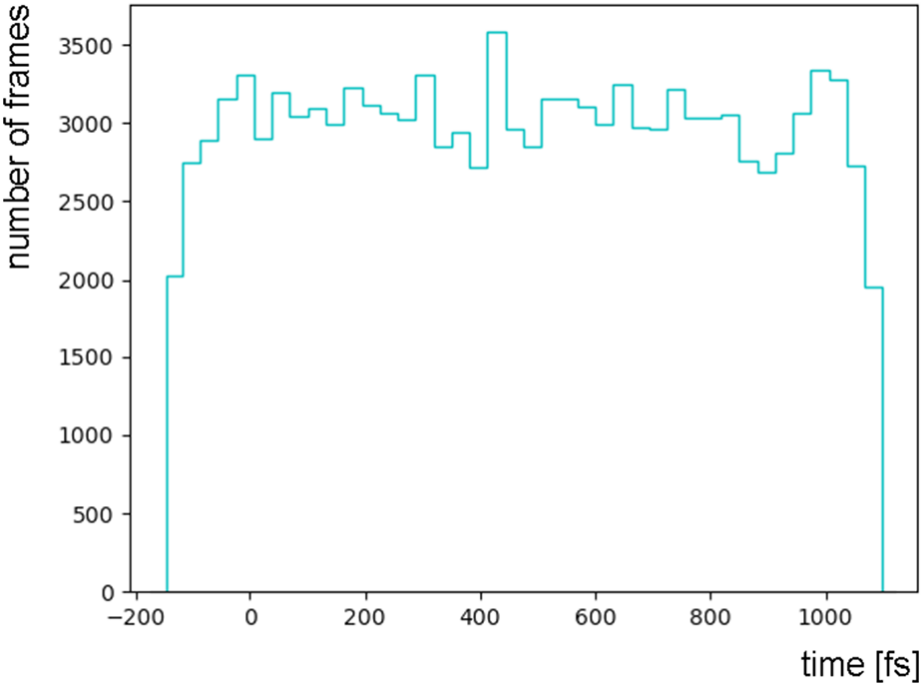
Distribution of pump-probe delay times of 119507 bacteriorhodopsin diffraction frames used as input to LPSA.

From the pump-probe delay distribution of 206 181 light-activated frames, we randomly select and discard frames, with the purpose of obtaining an approximately uniform distribution over time. We obtain a dataset comprised of *S* = 119 507 light-activated frames, covering roughly the first picosecond after photoactivation (Fig. 1).

The probability of observing a certain reflection is not homogeneous throughout the data matrix but rather decreases with increasing resolution, that is, with increasing modulus of the corresponding transferred wavevector *Q*. To account for this fact and following Ref. 16, we apply a redundancy-based scaling. We divide each intensity value by the number of times the corresponding (*h*, *k*, *l*) triplet is observed across the entire dataset. The fact that this scaling is necessary and appropriate is demonstrated in Sec. IV C.

### C. LPSA results

LPSA involves two main parameters, the concatenation number *q* and the cutoff value $j_{\text{max}}$, defining the maximal frequency $j_{\text{max}}\omega$ employed to build the projector's basis functions. Other parameters involved are the number of modes $\tilde{r}$ employed in the dynamics reconstruction and the number of copies 2p+1 over which the averaging of reconstructed data vectors is performed. We show in supplementary material Fig. 1 that the value of *p* has little impact on the quality of the reconstruction. Hence, in what follows we adopt *p* = 0, to reduce the computational cost.

#### 1. Parameter optimization strategy

To select an appropriate $\left(q,j_{\text{max}},\tilde{r}\right)$ set, we carry out parameter scans. We calculate the singular-value spectra of the reduced data representation ***A*** with a fixed value of *q* and varying $j_{\text{max}}$, and, in turn, with fixed $j_{\text{max}}$ and variable *q*. Similarly, we calculate the indicator *L*, whose definition is introduced in Ref. 13 and reported hereafter for completeness, quantifying the deviation from local linearity of the reconstructed trajectory, as a function of the number of modes employed. We denote with $\left\{x_{j}\right\}$ the sequence of time-ordered reconstructed data vectors related to the time points $\left\{t_{j}\right\}$, with $x_{j}=x(t_{j})$. From any pair of temporally neighboring reconstructed data vectors, $x_{j-1}$ and $x_{j}$, we can construct a local linear approximation to x(t), which we call $x^{\left(j\right)}(t)$,

$$x^{\left(j\right)}(t)=x_{j-1}+\frac{t-t_{j-1}}{t_{j}-t_{j-1}}(x_{j}-x_{j-1}).$$ (12)

Local linearity implies that the two immediate temporal neighbors of $x_{j-1}$ and $x_{j}$, i.e., $x_{j-2}$ and $x_{j+1}$, lie close to the points $x^{\left(j\right)}(t_{j-2})$ and $x^{\left(j\right)}(t_{j+1})$, respectively. We, therefore, define

$$L^{\left(j\right)}=\frac{1}{2}\left[\left|x_{j-2}-x^{\left(j\right)}(t_{j-2})\right|+\left|x_{j+1}-x^{\left(j\right)}(t_{j+1})\right|\right].$$ (13)

The average over all $L^{\left(j\right)}$ represents our measure of deviation from local linearity *L*.

In favorable cases, these indicators allow one to select the appropriate number of modes to be employed in the reconstruction. A sharp decrease in the spectra when a certain value of $\tilde{r}$ is exceeded indicates that additional modes make a negligible contribution to the reconstruction. A concomitant increase in *L* indicates that modes beyond the $\tilde{r}$th contribute mainly noise to the reconstructed trajectories (see Ref. 13). Once the number of modes is selected, the convergence of the solution can be assessed by calculating, with fixed $j_{\text{max}}$, the correlation coefficients between pairs of successive solutions obtained by progressively increasing *q* and, in turn, with fixed *q*, the correlation between solutions obtained by increasing $j_{\text{max}}$.

#### 2. Parameter optimization results

**FIG. 2.**
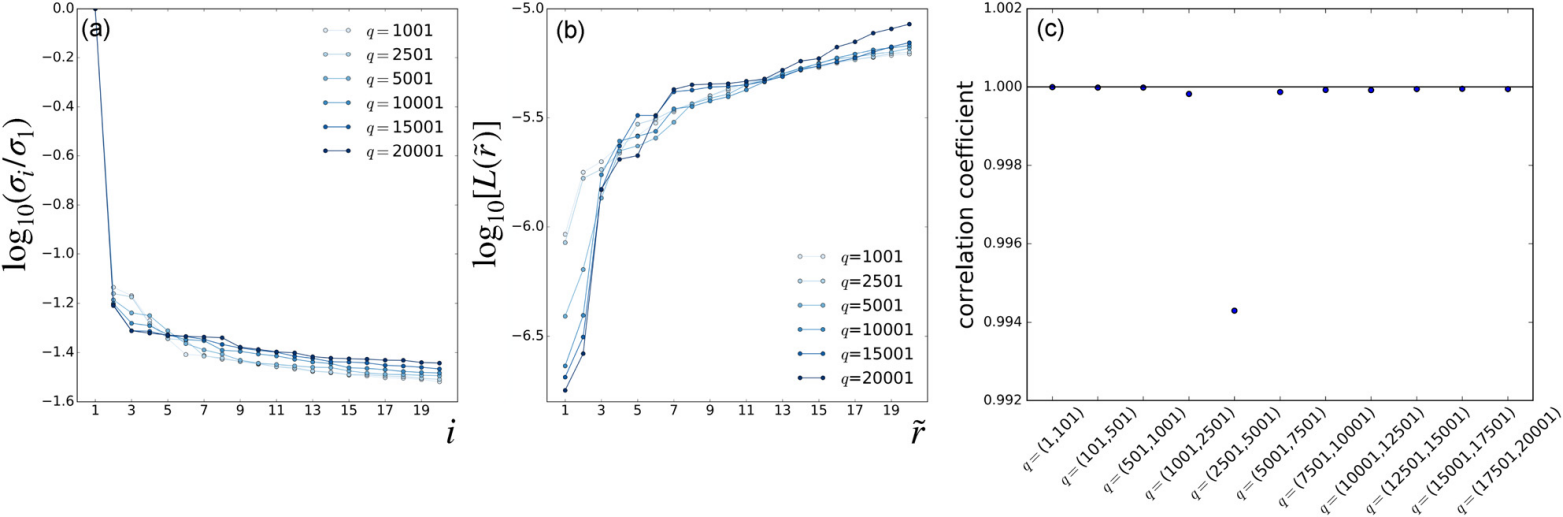
LPSA of bR TR-SFX data. LPSA *q*-scan with $j_{\text{max}}=20$. (a) Singular-value spectra. (b) Deviation from local linearity of the solution with *p* = 0 as a function of the number of modes employed $\tilde{r}$. (c) Correlation coefficient between successive solutions obtained with increasing *q*, calculated with *p* = 0 and $\tilde{r}=2$ modes.

**FIG. 3.**
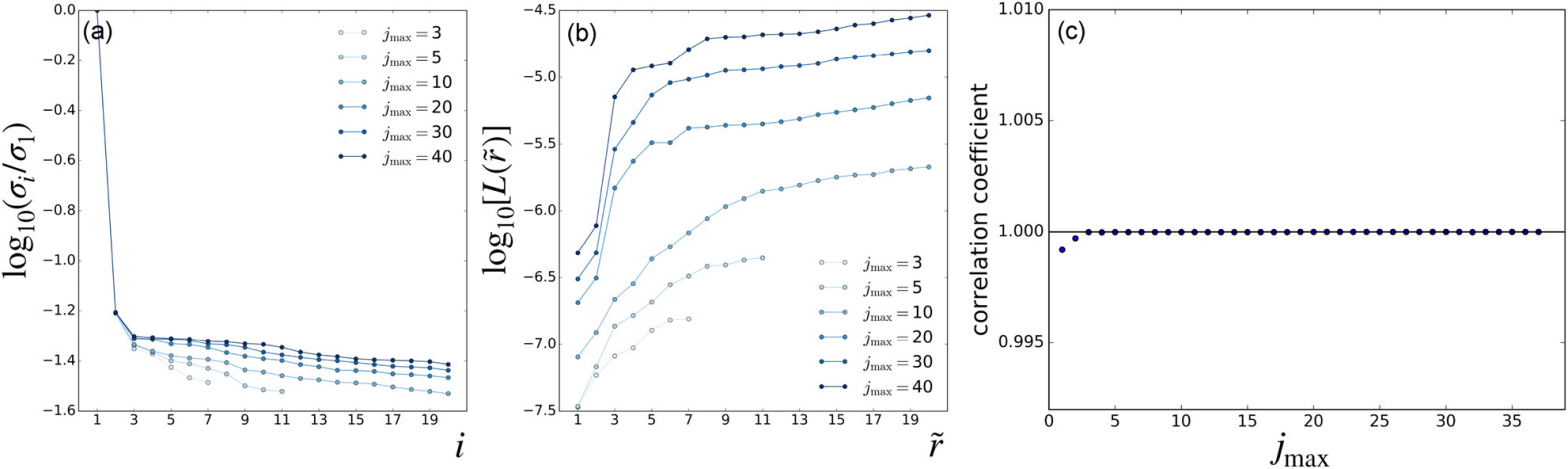
LPSA of bR TR-SFX data. LPSA $j_{\text{max}}$-scan with *q* = 15 001. (a) Singular-value spectra. (b) Deviation from local linearity of the solution with *p* = 0 as a function of the number of modes employed $\tilde{r}$. (c) Correlation coefficient between successive solutions obtained with $j_{\text{max}}$ and $j_{\text{max}}+1$, calculated with *p* = 0 and $\tilde{r}=2$ modes.

Figures 2 and 3 present the results of parameter scans from the LPSA of the bR TR-SFX dataset. The singular-value spectra of ***A*** and the indicator *L* as a function of the number of modes employed are shown in Figs. 2(a) and 2(b), respectively, using a fixed $j_{\text{max}}=20$ and varying the value of *q*. Similarly, Figs. 3(a) and 3(b) present spectra and deviation from local linearity, respectively, now using varying values of $j_{\text{max}}$ with fixed *q* = 15001. Singular-value spectra consistently show that all modes after the first one have a similar weight [Figs. 2(a) and 3(a)]. This means that, for a given $\left(q,j_{\text{max}}\right)$ set, no convergence with respect to the number of modes employed in the reconstruction can be expected, since each additional mode introduces new features with a weight similar to the previous one.

**FIG. 4.**
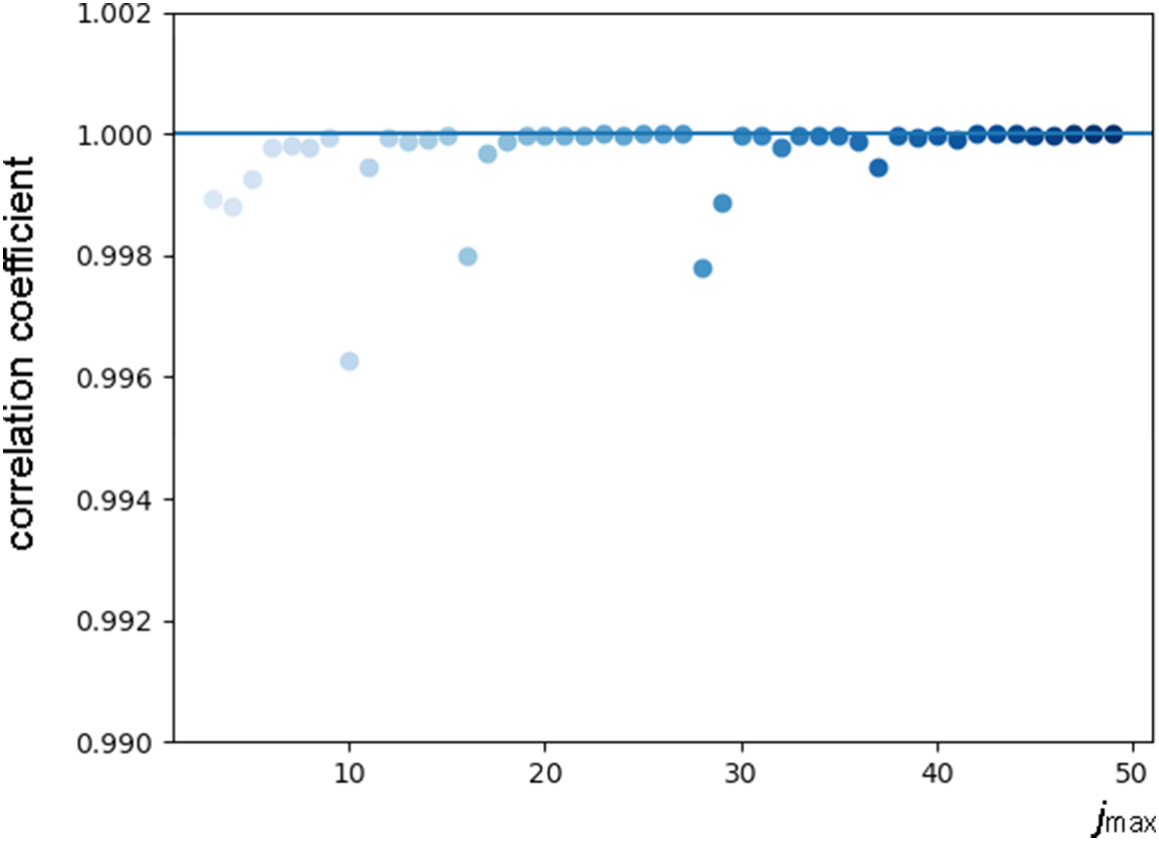
LPSA of bR TR-SFX data with *q* = 15001. Correlation coefficient between successive solutions obtained with $j_{\text{max}}$ and $j_{\text{max}}+1$, calculated with *p* = 0 and 6 modes.

Using as a guide the end of the plateau section in the deviation from local linearity observed in Fig. 3(b), we adopt the tentative value $\tilde{r}=6$ and calculate the correlation between successive solutions obtained by increasing $j_{\text{max}}$ in steps of one (Fig. 4). Dips in correlation correspond to new features adding up to the solution when increasing $j_{\text{max}}$ by one unit and are expected to occur when $j_{\text{max}}$ lies below the high-frequency end of the dynamical spectrum of the system. Unexpectedly, correlation dips are observed up to $j_{\text{max}}=38$, corresponding to physically implausible high frequencies.

**FIG. 5.**
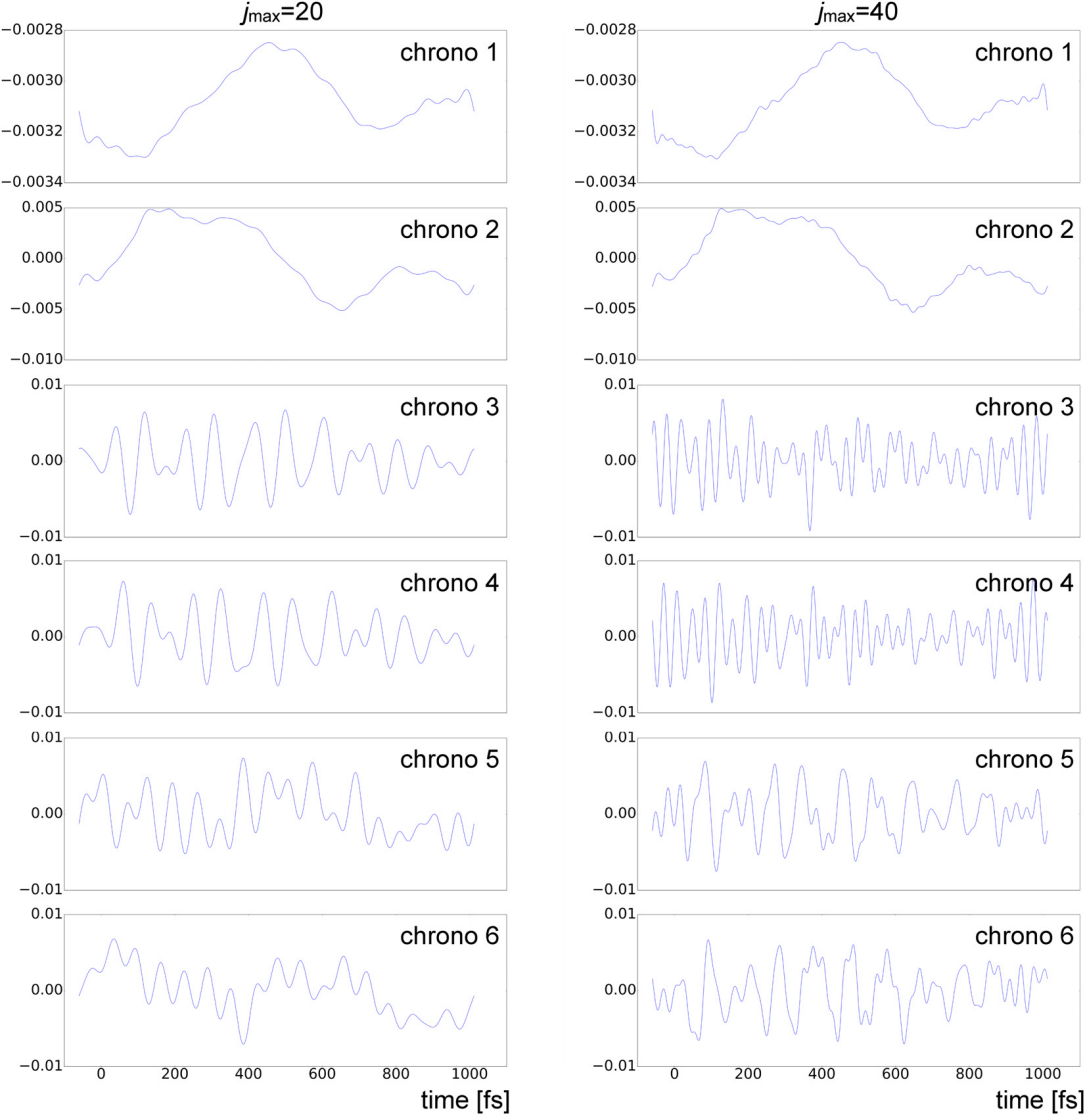
Chronograms from LPSA of bR TR-SFX data with *q* = 15 001; $j_{\text{max}}=20$ (left) and $j_{\text{max}}=40$ (right).

Inspection of LPSA chronograms $w_{i}$ helps to clarify the issue. We observe that low-order modes are contaminated by high frequencies, corresponding to periods down to a few tens of femtoseconds, which are not physically sound, considering the long duration of the pump pulse ( ∼100 fs FWHM). Figure 5 shows that all modes after the first two are dominated by such spurious features. As can be observed by comparing chronograms 3–6 at different $j_{\text{max}}$ values, these high-frequency features are $j_{\text{max}}$-dependent, which explains the lack of convergence at plausible $j_{\text{max}}$ values observed in Fig. 4.

We, nevertheless, observe that the first two bR LPSA modes are barely affected by high-frequency contamination (Fig. 5). They also appear to be stable with varying $j_{\text{max}}$. In addition, the deviation from local linearity *L* as a function of the number of modes shows a large increase after the second mode [Figs. 2(b) and 3(b)]. By selecting only the first two modes, the solution appears to be stable both as a function of *q* [Fig. 2(c)] and as a function of $j_{\text{max}}$ [Fig. 3(c)]. We call this a *quasi-*solution because convergence with respect to $\tilde{r}$ is not fulfilled. Difference-electron-density maps calculated using these two modes (see map calculation details in Sec. V) show isomerization features compatible with previous findings (supplementary material Fig. 2).

In Sec. IV, we use a synthetic dataset to investigate the root cause of high-frequency contamination of LPSA modes. Using model data, we can isolate different sources of stochasticity, which are known to affect TR-SFX data and analyze their impact on dynamics retrieval.

## IV. DYNAMICS RETRIEVAL FROM SYNTHETIC DATA AFFECTED BY STOCHASTICITY

**FIG. 6.**
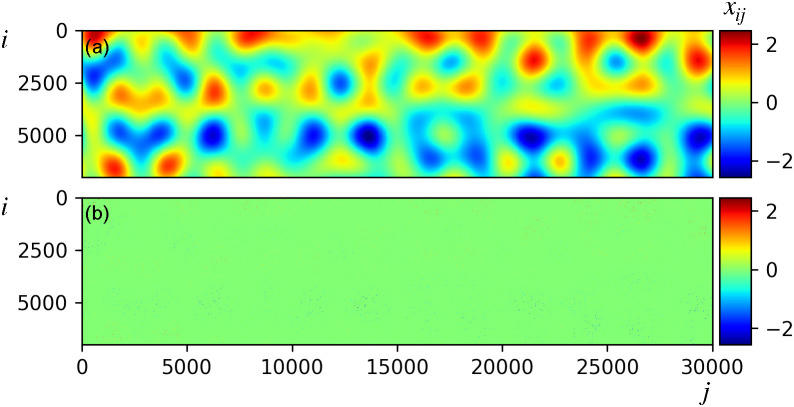
(a) Underlying dynamics x(t) [Eq. (14)], with *x_ij_* the *i*-th component of data vector $x_{j}=x(t_{j})$. Here, *m* = 7000 and *S* = 30 000. (b) Incomplete (98.2% missing observations) and partial input data. Missing entries are assigned zero values generating a sparse input data matrix.

In this section, we seek to identify the root cause of the behavior observed in our TR-SFX dataset, particularly that of the singular-value spectra as well as the high-frequency contamination of the chronograms. To this end, we use a model dynamical system to investigate the impact of various sources of stochastic errors, which are known to be relevant in TR-SFX datasets, individually and in combination. We employ the same model as was used in Ref. 13,

$$\begin{array}{c} x(t)=(1-e^{\left(-t/t_{c}\right)})[A+B\;\text{cos}\;(3\omega t)+C\;\text{sin}\;(10\omega t)]\\ +e^{\left(-t/t_{c}\right)}[D+E\;\text{sin}\;(7\omega t)+F\;\text{sin}\;(11\omega t+\pi /10)], \end{array}$$ (14)

shown in Fig. 6(a), with *t_c_* corresponding to the middle of the time interval considered, ***A***, ***B***, ***C***, ***D***, ***E***, and ***F*** noncollinear vectors $\in {\mathbb{R}}^{m}$, with components

$$\begin{array}{c} A_{i}=\text{cos}\;[0.6\chi i],\\ B_{i}=\text{sin}\;[3.0\chi i+\pi /5],\\ C_{i}=\text{sin}\;[0.8\chi i+\pi /7],\\ D_{i}=\text{cos}\;[2.1\chi i],\\ E_{i}=\text{cos}\;[1.2\chi i+\pi /10],\\ F_{i}=\text{sin}\;[1.8\chi i+\pi /11] \end{array}$$ (15)

for i=1,…,m and χ=2π/m.

Stochastic weighting (partiality) and extreme data incompleteness, with a level of 98.2% missing observations distributed homogeneously in space and time, were included in the model studied in Ref. 13. Partiality was modeled by multiplying each intensity value by a random number extracted from a uniform distribution comprised between zero and one. It was shown that LPSA can achieve excellent dynamics retrieval, superior to binning-and-merging, and similar to SSA and NLSA. Here, we study the impact of timing uncertainty (Sec. IV A), increasing homogeneous incompleteness and pixel-dependent incompleteness (Secs. IV B and IV C), and photon counting errors (Sec. IV D). Unless stated otherwise, missing observations are assigned a value of zero, mapping data incompleteness to matrix sparsity. In Sec. IV E, we show that a modified handling of missing observations is required to obtain optimal results from datasets with a large static component.

### A. Timing uncertainty

Timing uncertainty represents an important challenge in TR-SFX. It is well known that once the delay line is adjusted to fulfill a certain pump-probe delay requirement, the effectively generated delay jitters considerably. Jitter values are generally large (100–200 fs root mean squared deviation^19^), and the determination of the pump-probe delay can be improved at the individual shot level, by using a timing-tool, which is normally characterized by a substantially smaller uncertainty (see, for example, Refs. 19 and 20). Here, we investigate how the extent of this residual stochastic error affects the quality of dynamics retrieval.

To simulate the random component of the pump-probe timing uncertainty, we introduce in our partiality- and sparsity-affected model data increasing levels of timing errors using the following procedure. We calculate model estimates at a set of homogeneously distributed, but not necessarily equally spaced, underlying time values ${\tilde{t}}_{j}$. To mimic the experimental error, we assign to each time point ${\tilde{t}}_{j}$ a corresponding *t_j_*, which is meant to represent the measured value and is extracted from a normal distribution centered on ${\tilde{t}}_{j}$ and with standard deviation $\sigma_{\text{jitter}}$. Frames are then ordered based on the values of *t_j_*. We express the timing error $\sigma_{\text{jitter}}$ in terms of fractions of $T_{\text{min}}$, the shortest period present in the dynamics [Eq. (14)].

We analyze datasets with 98.2% sparsity, partiality, and increasing timing error $\sigma_{\text{jitter}}$ in the range between 0.1 and $1.0T_{\text{min}}$. All tested jitter values introduce errors on the order of the frames. The rationale behind LPSA parameter selection is outlined in Sec. III C 1. As mentioned in Sec. III, *p* = 0 is employed in all reconstructions [Eq. (10)] because this is the computationally cheapest option, and the quality of the retrieved signal does not depend substantially on the choice of *p* (supplementary material Fig. 1).

**FIG. 7.**
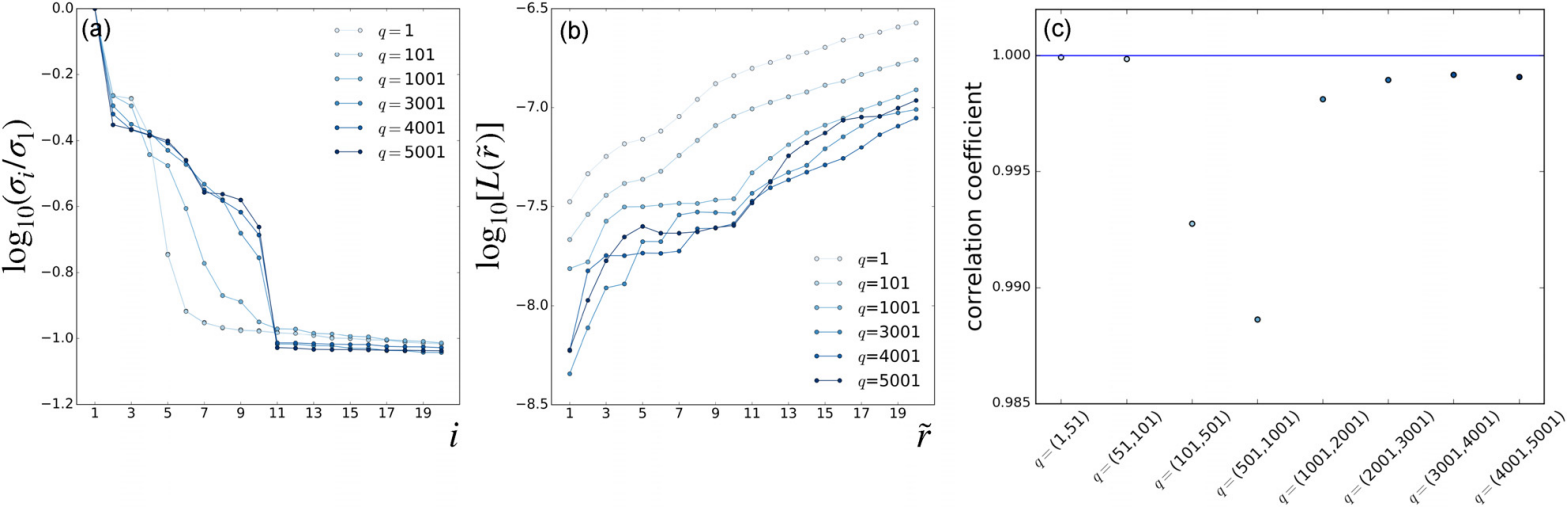
LPSA of synthetic data with sparsity (98.2%), partiality, and timing error $\sigma_{\text{jitter}}=0.1T_{\text{min}}$. LPSA *q*-scan with $j_{\text{max}}=100$. (a) Singular-value spectra. (b) Deviation from local linearity of the solution with *p* = 0 as a function of the number of modes employed $\tilde{r}$. (c) Correlation coefficient between successive solutions obtained with increasing *q*, calculated with $\tilde{r}=10$ modes and *p* = 0.

**FIG. 8.**
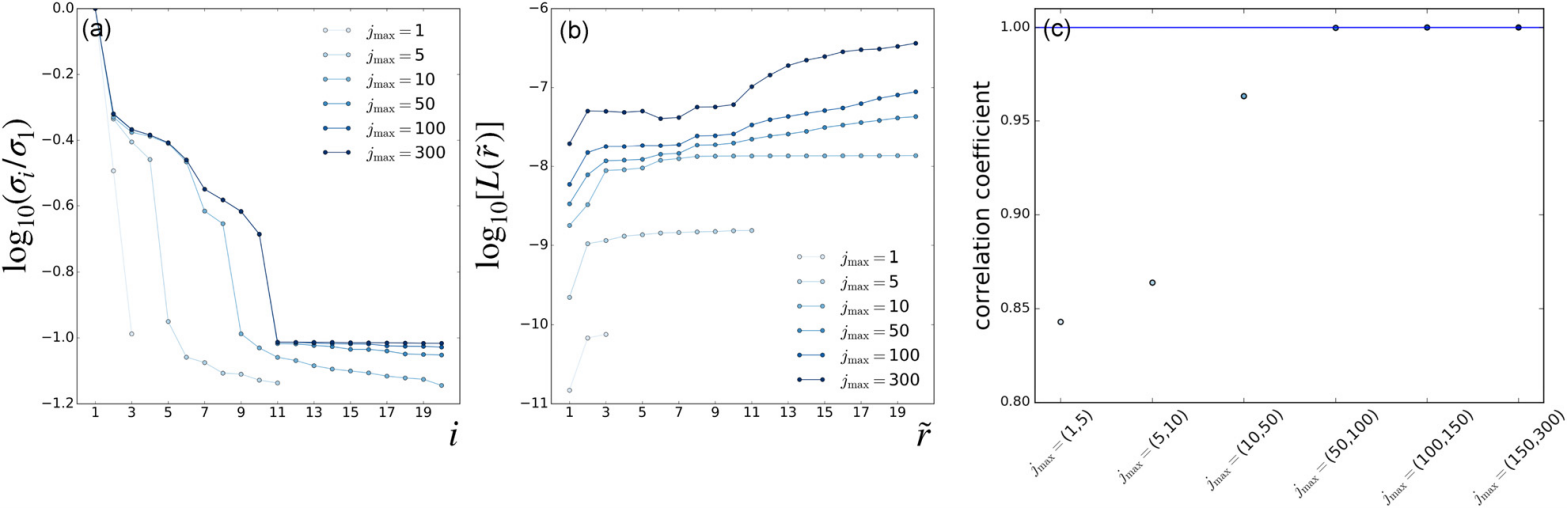
LPSA of synthetic data with sparsity (98.2%), partiality, and timing error $\sigma_{\text{jitter}}=0.1T_{\text{min}}$. LPSA $j_{\text{max}}$-scan with *q* = 4001. (a) Singular-value spectra. (b) Deviation from local linearity of the solution with *p* = 0 as a function of the number of modes employed $\tilde{r}$. (c) Correlation coefficient between successive solutions obtained with increasing $j_{\text{max}}$, calculated with $\tilde{r}=10$ modes and *p* = 0.

Figures 7 and 8 show the LPSA parameter selection procedure for our sparse and partial model data, with timing error $\sigma_{\text{jitter}}=0.1T_{\text{min}}$. Figures 7(a) and 7(b) present the singular-value spectra and the deviation from local linearity as a function of the number of modes $\tilde{r}$, for a set of low-pass spectral analyses performed by keeping a fixed value of $j_{\text{max}}=100$ and varying *q*. The concomitant sharp decrease in singular value and increase in deviation from local linearity when the number of modes exceed 10 indicate that modes beyond the 10th contribute mainly noise to the reconstruction. Figure 7(c) shows the correlations between successive solutions, obtained with $j_{\text{max}}=100$ and by progressively increasing the values of *q*. The solution appears to be relatively stable in the range between *q* = 2001 and *q* = 5001.

**FIG. 9.**
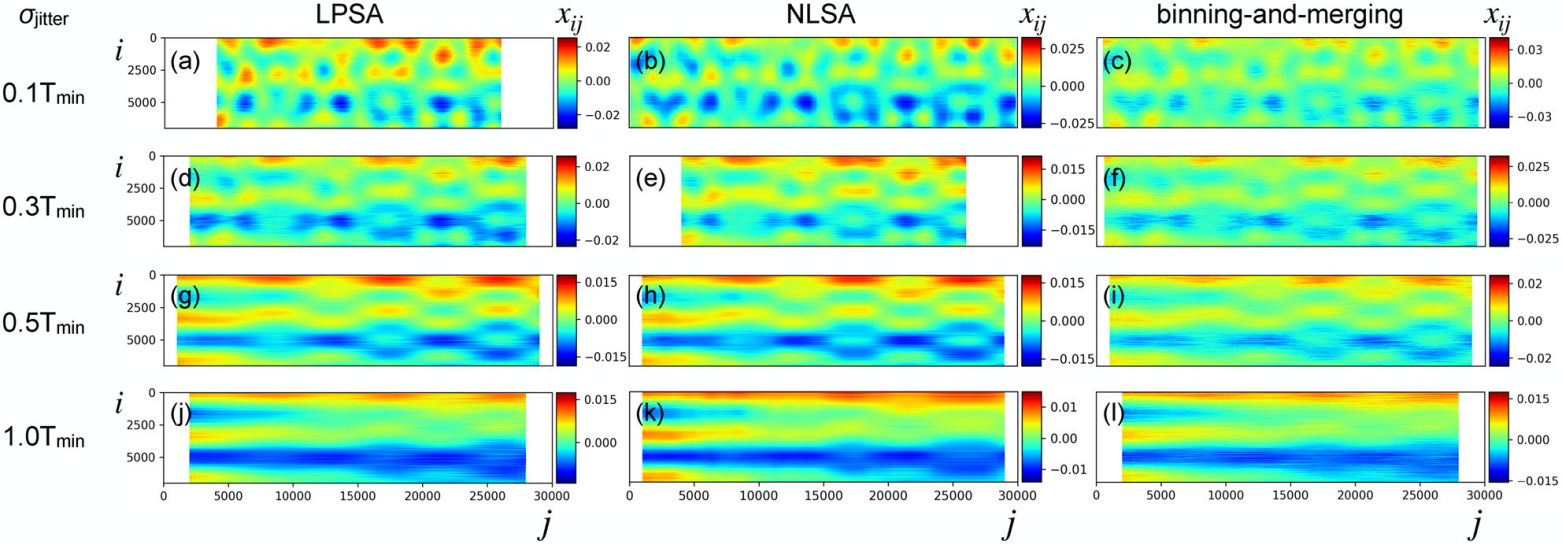
Synthetic data with sparsity (98.2%), partiality, and increasing levels of timing error with $\sigma_{\text{jitter}}$ equal to $0.1T_{\text{min}}$ [(a)–(c)], $0.3T_{\text{min}}$ [(d)–(f)], $0.5T_{\text{min}}$ [(g)–(i)], and $1.0T_{\text{min}}$ [( j)–(l)]. Dynamics retrieval, with optimized parameters, by LPSA [(a), (d), (g), and (j)], NLSA [(b), (e), (h), and (k)], and binning-and-merging [(c), (f), (i), and (l)]. Parameters used in the analyses and correlation between the retrieved dynamics and the ground truth are reported in Table I.

With *q* = 4001, we analyze the results obtained with varying $j_{\text{max}}$. Figures 8(a) and 8(b) show singular-value spectra and deviation from local linearity for various values of $j_{\text{max}}$. Consistent with Fig. 7, 10 modes appear to be an appropriate choice. Figure 8(c) shows the correlation coefficients between pairs of successive solutions obtained by increasing $j_{\text{max}}$ progressively and using 10 modes. We observe that the solution is stable in the $j_{\text{max}}$-range between 50 and 300. The set $\left(q=4001,j_{\text{max}}=100,\tilde{r}=10,p=0\right)$ is used to retrieve the dynamics, and the result is shown in Fig. 9(a).

**FIG. 10.**
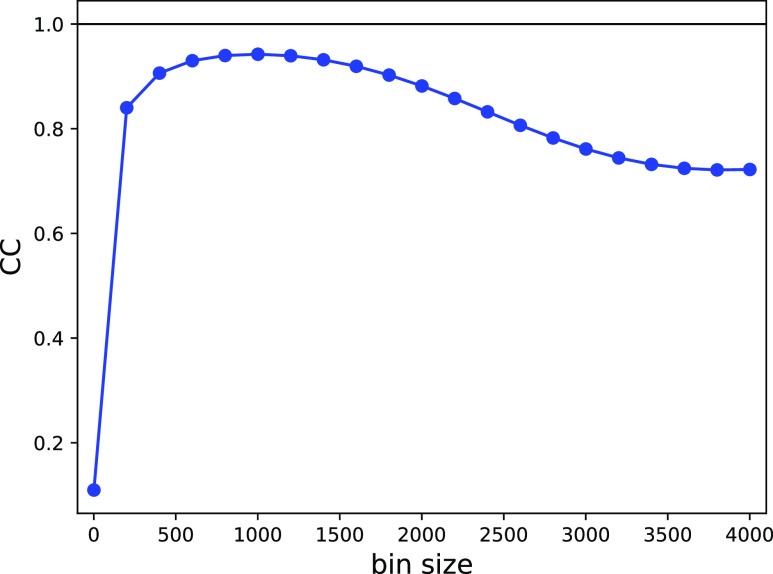
Binning-and-merging of synthetic data with sparsity (98.2%), partiality, and timing error $\sigma_{\text{jitter}}=0.1T_{\text{min}}$. Correlation coefficient to the ground truth as a function of the bin size.

For comparison, we also apply NLSA to our dataset.^15,16^ The results of NLSA-parameter optimization are shown in supplementary material Fig. 3. The quality of the retrieved dynamics, shown in Fig. 9(b), is found to be similar to that from LPSA. The same dataset is treated by binning-and-merging. For a fixed bin size, intensities from all frames within a sliding window of that size are averaged. The best bin size is determined by calculating the correlation between the sliding average and the ground truth (Fig. 10), a generally unavailable metric. The best binning-and-merging outcome is shown in Fig. 9(c). The reconstruction quality from binning-and-merging is inferior to that from LPSA and NLSA, with a correlation to the ground truth of 0.9424 compared to 0.9826.

Figure 9 presents a summary of the results obtained by progressively increasing the timing uncertainty in our dataset up to the value $\sigma_{\text{jitter}}=1.0T_{\text{min}}$. LPSA, NLSA, and binning-and-merging are carried out with optimized parameters, using model data with timing uncertainty of $0.1T_{\text{min}}$ [panels (a)–(c)], $0.3T_{\text{min}}$ [panels (d)–(f)], $0.5T_{\text{min}}$ [panels (g)–(i)], and $1.0T_{\text{min}}$ [panels (j)–(l)]. We observe that LPSA and NLSA produce very similar results in terms of quality of the retrieved dynamics. With relatively low values of $\sigma_{\text{jitter}}$ in the range between 0.1 and $0.5T_{\text{min}}$, LPSA and NLSA provide superior dynamics retrieval compared to binning-and-merging. The quality of the retrieval decays quickly with increasing timing uncertainty; with $\sigma_{\text{jitter}}=1.0T_{\text{min}}$, most dynamical features cannot be retrieved.

Although the quality of the retrieved dynamics is critically affected by increasing timing uncertainty, the main impact appears to be the smearing of dynamical features, provided that we operate in parameter space regions of solution stability. Importantly, we do not observe a contamination of the solution from spurious high-frequency features of the kind found in our experimental dataset. High-frequency contamination of the reconstructed dynamics can be observed in regions of parameter space lacking solution stability. This is recognizable particularly at low values of *q* (supplementary material Fig. 4).

### B. Data incompleteness

**FIG. 11.**
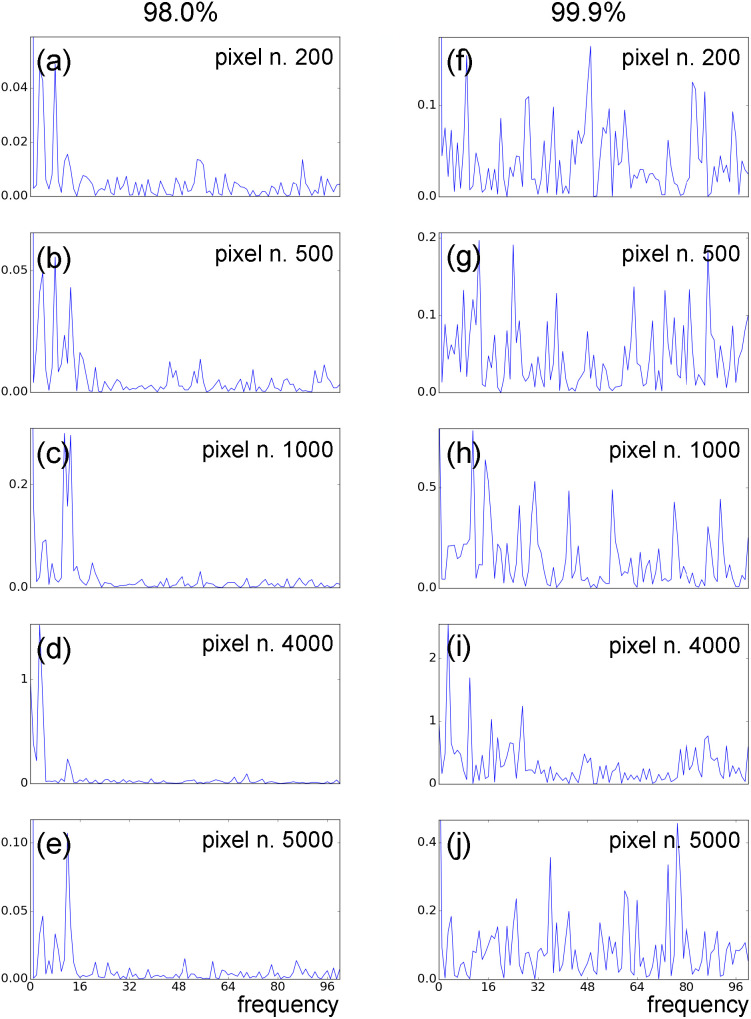
Fourier spectra of several traces $x_{i}(t)$ with *i* = 200 [(a) and (f)], *i* = 500 [(b) and (g)], *i* = 1000 [(c) and (h)], *i* = 4000 [(d) and (i)], and *i* = 5000 [(e) and (j)]. Data are affected by partiality, no timing uncertainty, and sparsity levels of 98% [(a)–(e)] and 99.9% [(f)–( j)].

We now introduce in our model data with partiality and no timing uncertainty, various levels of data incompleteness (matrix sparsity). Particularly, we compare input data with sparsity levels of 98% and 99.9%. Figure 11 shows the frequency spectra of the time evolution for some of the input data pixels. Spectral features related to the system's dynamics are clearly visible at the low-frequency end of the spectra for the 98% sparse data [Figs. 11(a)–11(e)]. An overlap between these features and low-frequency noise components can be observed for the 99.9% sparse dataset [Figs. 11(f)–11(j)]. Data sparsity affects the noise spectrum of the time evolution of the system. In particular, increasing sparsity generates a shift toward lower frequencies of the noise spectrum, producing an overlap between dynamical and noise spectral components.

**FIG. 12.**
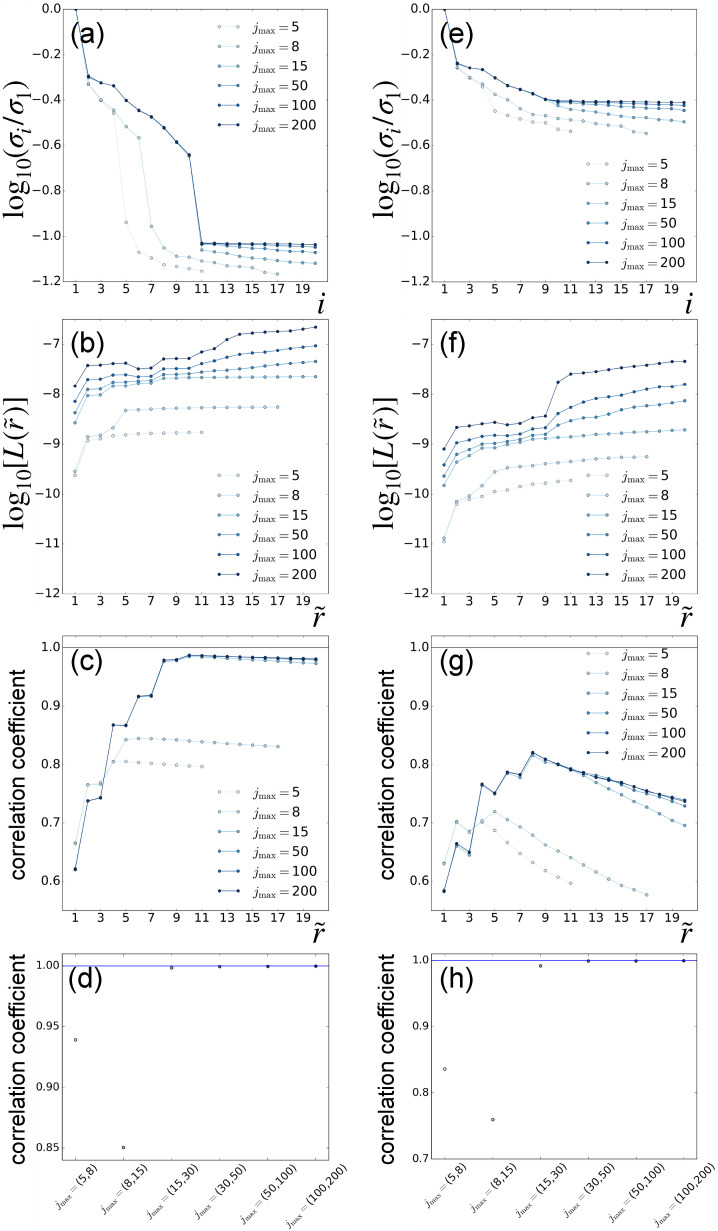
LPSA of synthetic data with partiality, no timing uncertainty, and sparsity levels of 98.0% [(a)–(d)] and 99.9% [(e)–(h)]. Parameter scan along the $j_{\text{max}}$-axis with fixed *q* = 4001. (a) and (e) Singular-value spectrum. (b) and (f) Deviation from local linearity of the solution with *p* = 0, as a function of the number of modes employed $\tilde{r}$. (c) and (g) Correlation coefficient of the solution with *p* = 0 with respect to the ground truth, as a function of the number of modes employed $\tilde{r}$. (d) and (h) Correlation coefficient between successive solutions calculated with increasing $j_{\text{max}}$, with $\tilde{r}=10$ and $\tilde{r}=9$ modes, respectively.

Figure 12 shows the LPSA singular-value spectra [panels (a) and (e)] and the deviation from local linearity as a function of the number of modes employed [panels (b) and (f)] for the two datasets described earlier, and with varying $j_{\text{max}}$. The singular-value spectra for the 99.9% sparse dataset do not show a decay to negligible values [panel (e)], a behavior that is similar to our TR-SFX dataset (Sec. III). Nevertheless, the singular-value spectra and the measure of deviation from local linearity [panels (e) and (f)] present a sharp decrease and increase, respectively, corresponding to $\tilde{r}=9$ modes. With this choice, the solution converges with respect to $j_{\text{max}}$ [panel (h)]. Inspection of the first 9 chronograms does not reveal a significant contamination from nonphysical high frequencies (supplementary material Fig. 5). Consistently, the solution obtained with optimized parameters $\left(q=4001,j_{\text{max}}=100,\tilde{r}=9,p=0\right)$ does not show any prominent spurious features (supplementary material Fig. 6). Compared to data with lower sparsity, the reconstruction quality deteriorates with higher sparsity, as the measurement of the correlation to the ground truth shows [Figs. 12(c) and 12(g)].

### C. Pixel-dependent data incompleteness

**FIG. 13.**
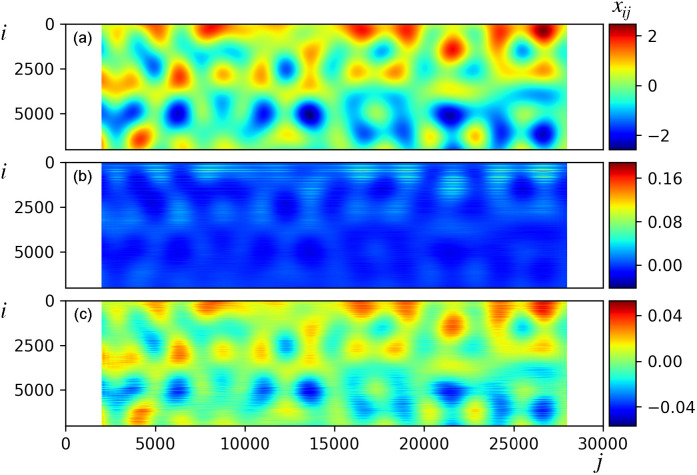
LPSA of synthetic data with partiality, no timing uncertainty, and pixel-dependent (time-independent) sparsity levels similar to those of TR-SFX datasets, ranging between 84.74% and 99.87%. (a) Ground truth. (b) Dynamics retrieval from LPSA with *q* = 4001, $j_{\text{max}}=100$, *p* = 0, and 10 modes. The correlation to the ground truth is 0.8183. (c) Dynamics retrieval from LPSA with *q* = 4001, $j_{\text{max}}=100$, *p* = 0, and 10 modes. Input data were subjected to redundancy-based scaling to account for the pixel-dependent sparsity level. The correlation to the ground truth is 0.9860.

TR-SFX data incompleteness is not homogeneous over reciprocal space, but rather it increases with resolution (or modulus of the transferred wavevector *Q*). To study the impact of this fact, we introduce in our partial model data with no timing uncertainty, a pixel-dependent sparsity level, with values comprised between 84.74% and 99.87%, to mimic the levels of data incompleteness found in our TR-SFX dataset. LPSA of these input data shows a rather poor quality of dynamics retrieval [Fig. 13(b)].

To account for the effect of pixel-dependent sparsity, we apply the redundancy-based scaling procedure first presented in Ref. 16 and described hereafter. For each pixel i=1,…,m, we divide the measured intensities $x_{i}(t)$ by the number of times the (*N_i_*) pixel *i* is observed in the dataset. This has the effect of scaling pixels with respect to each other so as to compensate for the variability in the number of observations. The LPSA result obtained using these data preparation procedure is shown in Fig. 13(c), with a remarkable improvement over the result without scaling.

### D. Gaussian noise

Although photon counts are known to follow a Poisson distribution, for simplicity of implementation, we mimic this effect by considering Gaussian errors in our model dataset. Such an approximation is in most cases well justified, as a Gaussian distribution approximates closely a Poisson distribution for intensity expectation values exceeding ten photons. First, using partial data with pixel-dependent sparsity and no timing uncertainty, we introduce Gaussian noise. Then, we repeat the same procedure using model data that include a pixel-dependent static component (pedestal),

$$I_{i}(t)=x_{i}(t)+C+i/C\prime ,$$ (16)

where $x_{i}(t)$ is given by Eq. (14), *i* is the pixel index ranging from 1 to *m* = 7000, *C* = 3, and various values of C′ are used to yield a small (C′=1000), medium (C′=600), and large (C′=250) static component. Individual observations are affected by Gaussian noise, with a standard deviation $\sigma ={\left|J_{i}(t)\right|}^{1/2}$, where $J_{i}(t)$ is obtained from $I_{i}(t)$ by considering the effects of data incompleteness (sparsity) and partiality. By including a time-independent contribution, we account for the fact that TR-SFX data present a relatively large static component and a relatively small dynamic one, since most atoms contributing to the structure factors do not move significantly over the time scales examined. The value of the static component is pixel-dependent, to mimic the fact that diffraction intensities tend to decrease with increasing resolution.

**FIG. 14.**
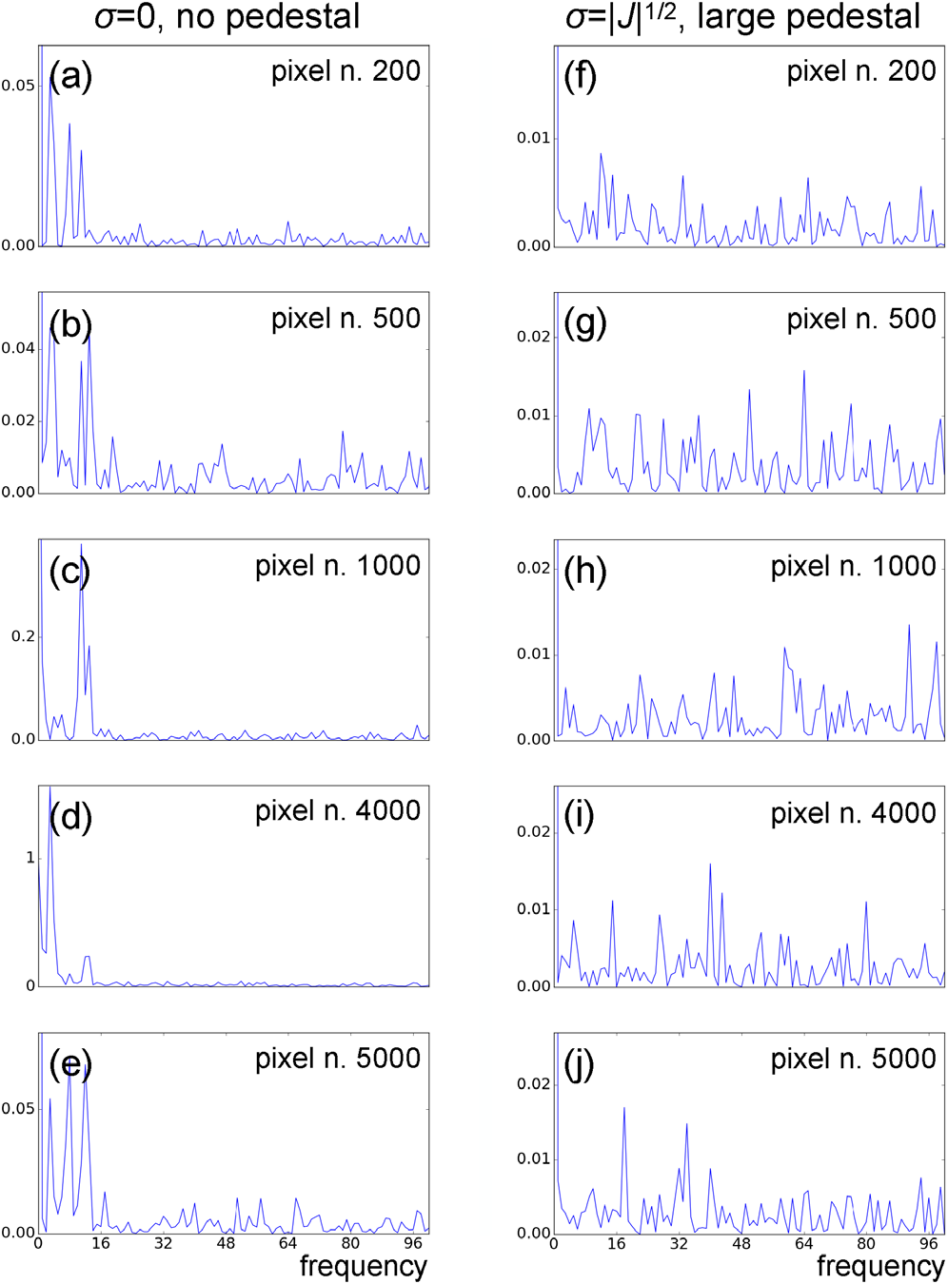
Fourier spectra of several traces $x_{i}(t)$ with *i* = 200 [(a) and (f)], *i* = 500 [(b) and (g)], *i* = 1000 [(c) and (h)], *i* = 4000 [(d) and (i)], and *i* = 5000 [(e) and (j)]. Data are affected by ***Q***-dependent sparsity, partiality, and no timing uncertainty. (a)–(e) Data with no pedestal and no noise. (f)-(j) Data with large pedestal and Gaussian noise with $\sigma ={\left|J\right|}^{1/2}$.

Figure 14 shows the Fourier spectra of the time traces of a few individual pixels from model data with partiality, pixel-dependent sparsity, and no timing uncertainty. Panels (a)–(e) show spectra from data with no static component and in the absence of Gaussian noise (*σ* = 0). Panels (f)–(j) show the Fourier spectra of the same pixels, now from data with a large static component, and $\sigma ={\left|J\right|}^{1/2}$. While low-frequency dynamical features are clearly visible in the first dataset, spectral components of the dynamics are completely overwhelmed by noise features in the second dataset. LPSA singular-value spectra for the two datasets are shown in supplementary material Fig. 7. We observe that while the first dataset presents a clear decay of the relative weights of the modes after the tenth [panel (a)], in the second dataset, all modes after the first one have a similar weight [panel (b)]. This can be attributed to noise features dominating the dynamics.

**FIG. 15.**
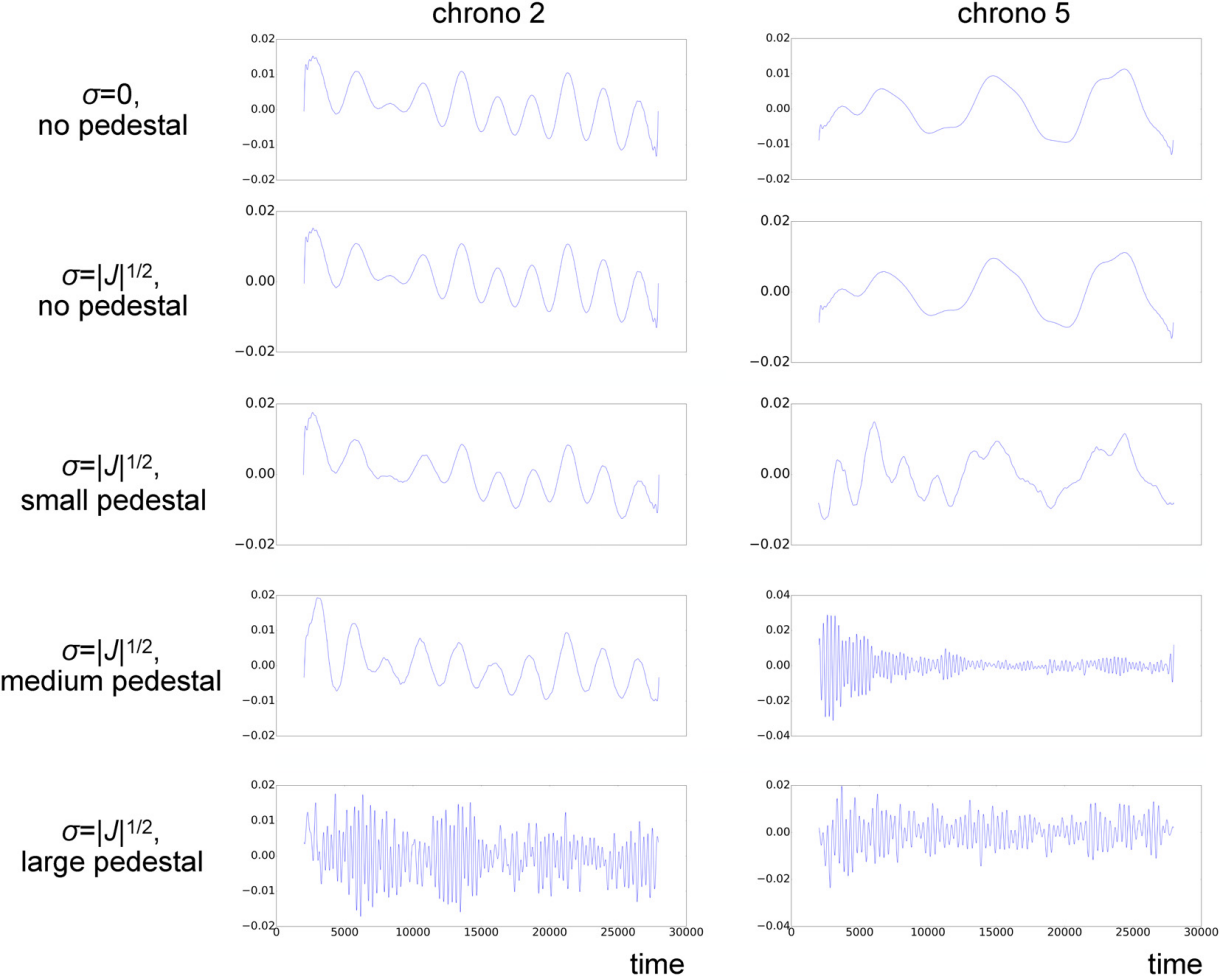
Chronograms from LPSA of synthetic data with ***Q***-dependent sparsity, partiality, no timing uncertainty, Gaussian noise *σ*, and various levels of pedestal. LPSA was performed with *q* = 4001 and $j_{\text{max}}=100$.

Figure 15 presents the evolution of some of the chronograms with increasing static component and Gaussian noise (see supplementary material Fig. 8 for a complete version, including the first 10 modes). Clearly, high-frequency, spurious features contaminate the chronograms when a larger static contribution is included, and Gaussian noise is considered. As noise grows with $\sigma ={\left|J\right|}^{1/2}$, an increase in the static component produces an increase in the amplitude of noise, while the size of the time-dependent component of the signal stays constant. With a large pedestal, low-frequency noise components overwhelm the components originating from the system dynamics, in the frequency spectra of the data, hampering the separation of physical features from noise.

### E. Treatment of missing observations

**FIG. 16.**
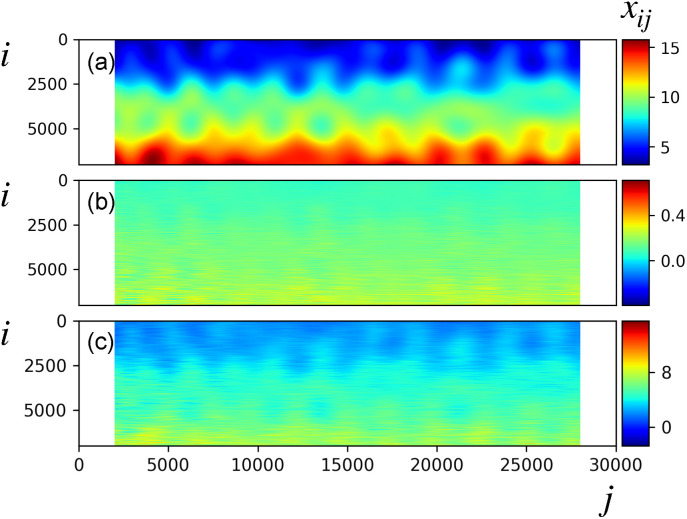
LPSA of data with partiality, pixel-dependent sparsity, and pixel-dependent static component (pedestal) 3+i/600, with i=1,…,m. (a) Underlying dynamics (ground truth). (b) LPSA of intensities according to procedure 1, with parameters *q* = 4001 and $j_{\text{max}}=100$. Reconstruction with *p* = 0 and 7 modes. CC to the ground truth: 0.9017. (c) LPSA of intensity deviations following procedure 2 (Sec. IV E), with parameters *q* = 4001 and $j_{\text{max}}=100$. Reconstruction with *p* = 0 and summation of the first 9 modes to the average. CC to the ground truth: 0.9608.

The analysis of data with a substantial static component reveals a suboptimal dynamics retrieval even in the absence of Gaussian noise. To exemplify this fact, we present the LPSA of synthetic data with a pixel-dependent static component, pixel-dependent incompleteness and partiality, and with no timing uncertainty and no Gaussian errors. In the procedure adopted so far (Refs. 13 and 16) which we now call procedure 1, unmeasured entries are assigned a value of zero, and intensities are weighted to account for the pixel-dependent redundancy (Sec. IV C) prior to LPSA. Using procedure 1, we observe high-frequency contamination of the chronograms (supplementary material Fig. 9) from the LPSA of data with a large static component. In addition, the time-dependent components of the retrieved dynamics appear to be suppressed in comparison to the static one [Fig. 16(b)].

A modification of the treatment of missing observations (procedure 2) allows to address both issues. Rather than assigning a value of zero to missing data matrix entries, we now compute the time average $\bar{x_{i}}$ of the *N_i_* existing measurements of pixel *i*. We assign this average value to missing observations. To emphasize the dynamical component, we subtract the pixel-dependent average $\bar{x_{i}}$ from all (*S*) observations of that pixel. The resulting intensity deviation matrix is highly sparse. While unmeasured entries are assigned to zero values, similar to procedure 1, measured values now represent intensity deviations from the average. This procedure allows to reduce the strong discontinuities in the input data, which are generated by procedure 1, when a large static component is present in the data. The immediate effect of this is the mitigation of the high-frequency contamination of the chronograms (supplementary material Fig. 9).

We account for the pixel-dependent incompleteness of the data and, at the same time, we scale the sparse intensity deviation matrix to the previously subtracted static component, by left-multiplication of the intensity deviation matrix by the diagonal matrix with elements $S/N_{i}$, prior to LPSA. The dynamics of the system is retrieved by adding the summation of the $\tilde{r}$ relevant LPSA modes to the average,

$$x(t)=\bar{x}+\sum_{j=1}^{\tilde{r}}\alpha_{j}(t)u_{j}.$$ (17)

The results of procedure 2 are shown in Fig. 16(c), with a remarkable improvement over procedure 1.

## V. LPSA OF A TR-SFX DATASET WITH AN IMPROVED TREATMENT OF MISSING OBSERVATIONS

Because of the limited extent of the structural changes happening on subpicosecond timescales, and the typically small excitation fraction, i.e., most molecules in the crystals remain unpumped, TR-SFX data are expected to include a large static component and a relatively small time-dependent contribution. We, therefore, present the LPSA of the bR TR-SFX dataset introduced in Sec. III, now handling missing observations according to procedure 2 (Sec. IV E), to optimally deal with data presenting a significant static component.

### A. Parameter optimization results

**FIG. 17.**
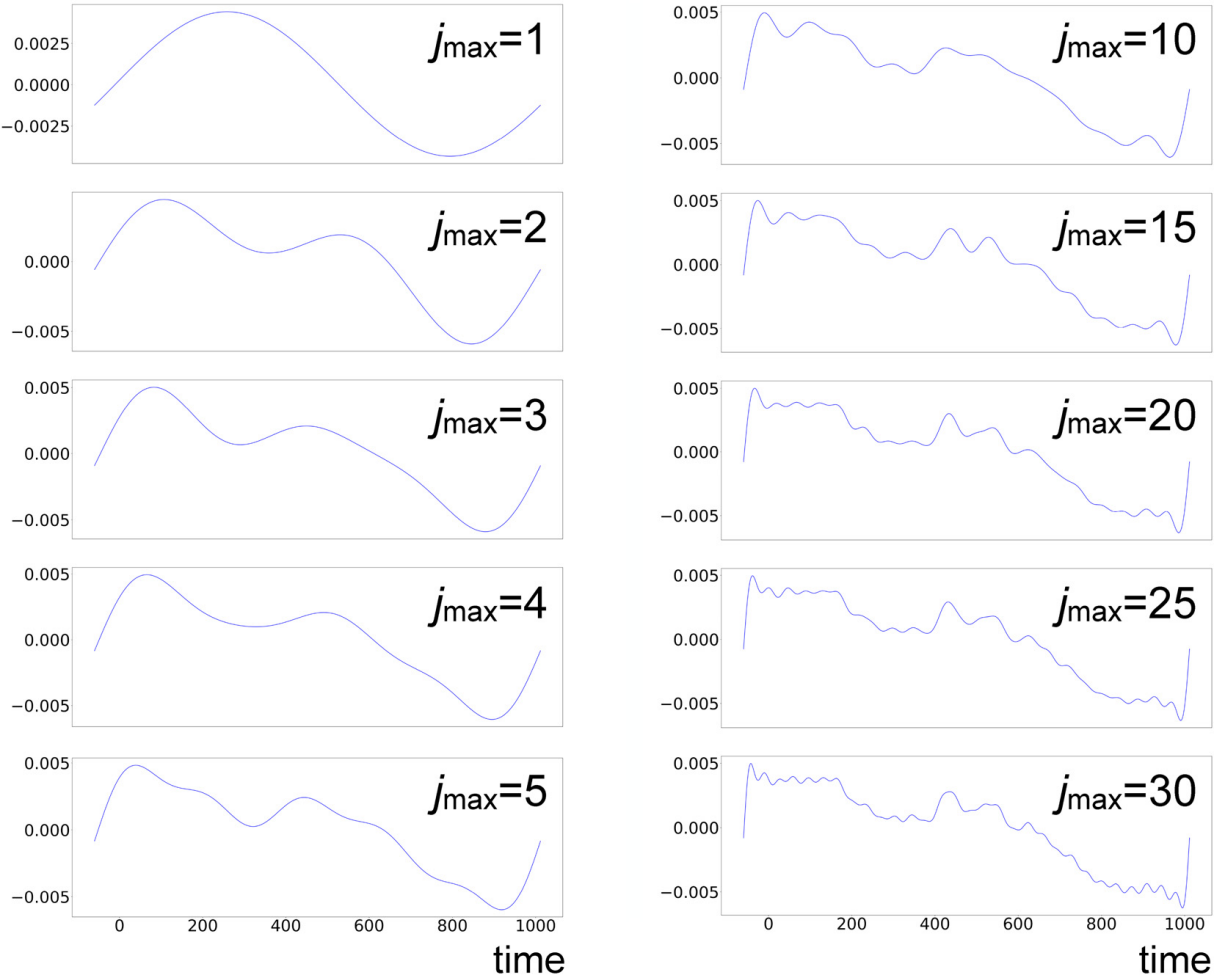
LPSA of bR TR-SFX data. Chronogram 1 with *q* = 15 001 and increasing $j_{\text{max}}$.

**FIG. 18.**
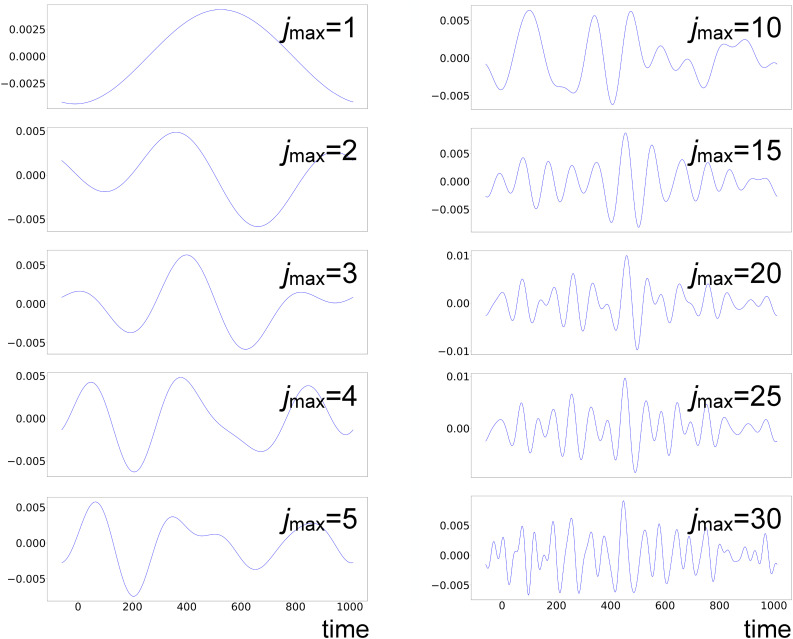
LPSA of bR TR-SFX data. Chronogram 2 with *q* = 15 001 and increasing $j_{\text{max}}$.

**FIG. 19.**
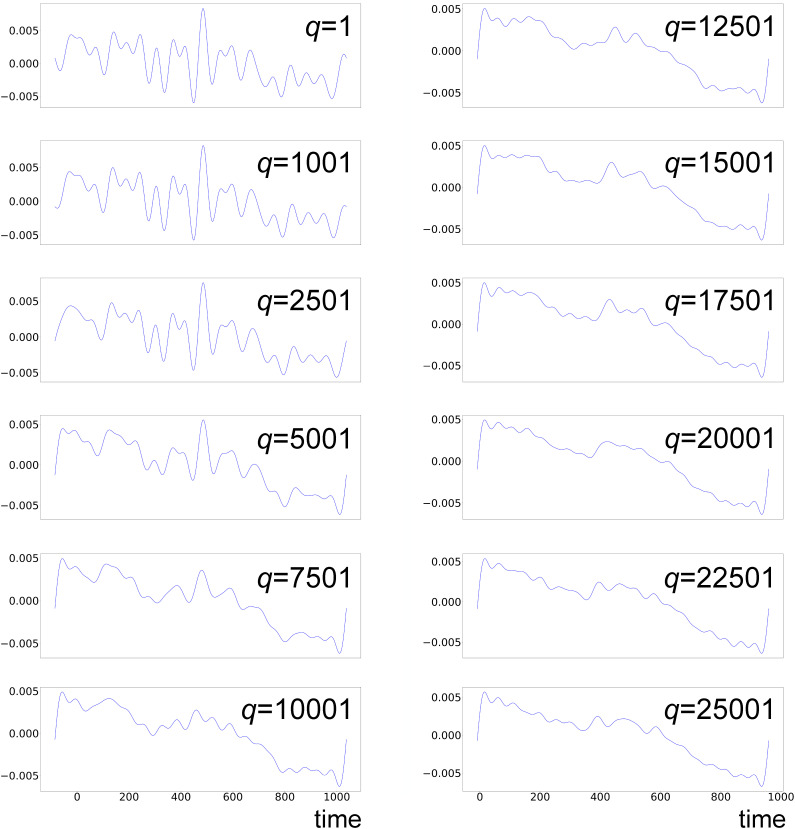
LPSA of bR TR-SFX data. Chronogram 1 with $j_{\text{max}}=20$ and increasing *q.*

We calculate intensity deviations according to procedure 2 (Sec. IV E) and carry out LPSA parameter scans. To discriminate between physical features and artifacts, we analyze the evolution of the leading chronograms as a function of the parameters involved. Figures 17 and 18 show the evolution of the first two chronograms with varying $j_{\text{max}}$ and fixed *q* = 15 001, while Fig. 19 shows chronogram 1 as a function of *q*, with fixed $j_{\text{max}}=20$. The time evolution of mode 1 (chronogram 1) is dominated by low-frequency features that persist with varying parameters. Low-amplitude, high-frequency oscillations are also present. These are parameter-dependent and identified as spurious. On the other hand, chronogram 2 does not show persistent oscillations with varying parameters. This indicates that chronogram 2 is overwhelmed by noise and needs to be discarded.

**FIG. 20.**
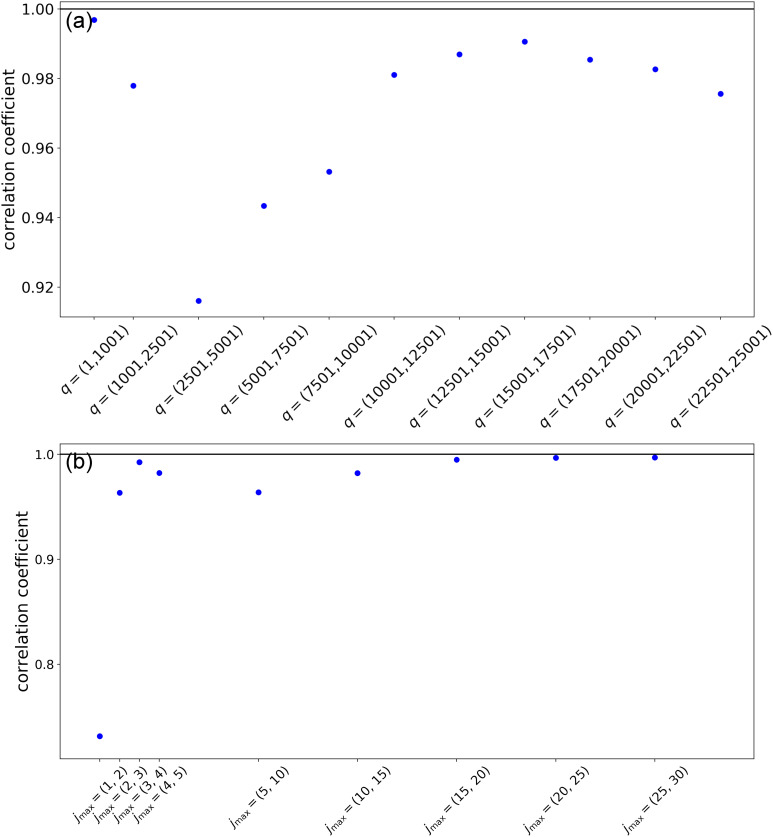
LPSA of bR TR-SFX data. Correlation coefficients between the first dynamical mode of successive solutions obtained with *p* = 0. (a) *q*-Scan with $j_{\text{max}}=20$. (b) $j_{\text{max}}$-Scan with *q* = 15 001.

Figure 20 shows the correlation coefficients between successive solutions obtained using only the first dynamical mode, with varying *q* and fixed $j_{\text{max}}=20$ [panel (a)] and varying $j_{\text{max}}$ with *q* = 15 001 [panel (b)]. The solution is relatively stable in the *q*-range around *q* = 15001 and with $j_{\text{max}}>15$. This is consistent with the observation that the first mode is dominated by low-frequency oscillations, which are conserved with varying parameters. The measurement of the deviation from local linearity *L* shows a large increase after the first mode [supplementary material Fig. 10(b)], suggesting that additional modes contribute mainly noise to the reconstruction.

We, therefore, consider the parameter set $q=15\;001,j_{\text{max}}=20,r=1$, and *p* = 0. Again, this is a *quasi-*solution because convergence with respect to *r* is not fulfilled. This is because successive modes have a similar weight (singular value) compared to the first one [supplementary material Fig. 10(a)]. While our analysis reveals that only one truly time-dependent mode can be recovered, i.e., r =1, $\bar{x}$ may be interpreted as the topogram of a static mode, and the retrieved dynamics [Eq. (17)] may be considered to involve two modes, similar to the results in Sec. III.

### B. Cyclic boundary conditions

**FIG. 21.**
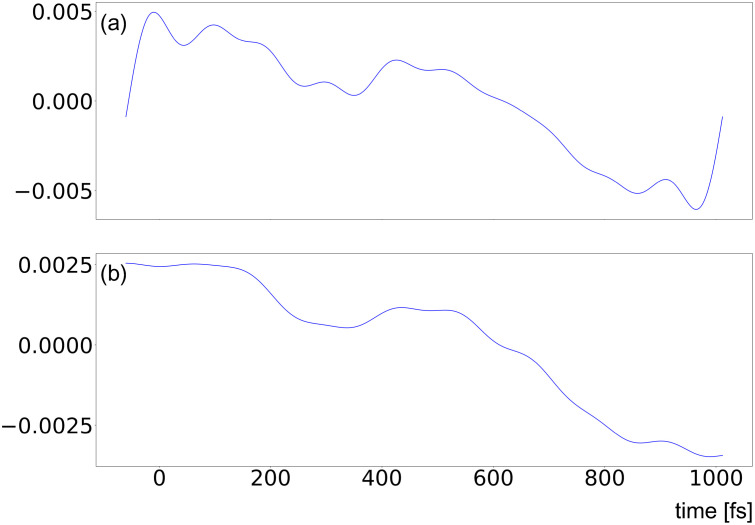
LPSA of bR TR-SFX data. (a) Chronogram 1 from the LPSA of ***x*** with *q* = 15 001 and $j_{\text{max}}=10$ and (b) from the LPSA of ***y*** with *q* = 15 001 and $j_{\text{max}}=20$. In (b), only the relevant time range is shown.

The LPSA formalism implies that the chronograms $w_{i}$, describing the time evolution of the dynamical modes, are a linear combination of orthonormalized trigonometric functions Φ. In addition to being continuous and differentiable, because the fundamental frequency corresponds to the time span in supervector space, these basis functions also fulfill cyclic boundaries conditions. While the assumption of smooth dynamics is physically sound, that of continuity at the boundaries is not in the dynamical system considered, where the final (isomerized) state of the molecule differs from the initial resting state. Imposing continuity at the boundaries in such a system results in high-frequency components being retained in the solution, which produce spurious rapid changes near the boundaries of the time range examined, and, possibly, contaminating high-frequency features in the entire time range [Fig. 21(a)].

To overcome these issues, we produce the following modified data matrix. Given the data $x\in {\mathbb{R}}^{m\times S}$, we build the matrix $y\in {\mathbb{R}}^{m\times 2S}$, with column vectors $y_{i}=x_{i}$, for i=1,…,S, and $y_{i}=x_{2S-i+1}$ for i=S+1,…,2S. By construction, when periodically repeated, $y_{i}$ is continuous at the boundaries. Reprocessing of the bacteriorhodopsin data according to this strategy allows to greatly mitigate artifacts in the resulting chronogram [Fig. 21(b)].

### C. TR-SFX LPSA quasi-solution

**FIG. 22.**
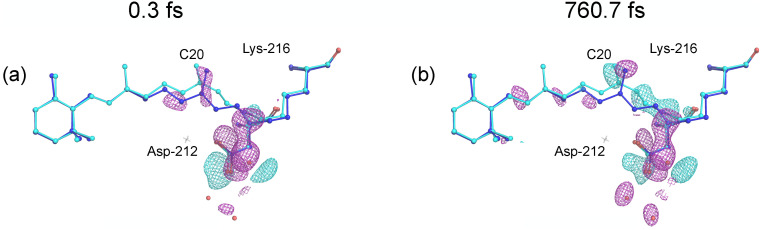
Stills from LPSA difference-electron-density movie obtained from bacteriorhodopsin TR-SFX data. Missing observations are treated according to procedure 2 (Sec. IV E), and LPSA of matrix ***y*** is performed with *q* = 15001 and $j_{\text{max}}=20$. The dynamics are reconstructed with *p* = 0 and using 1 mode, combined with the static component. Resting-state data are averaged. Maps are calculated following Ref. 10. Models of retinal chromophore and residues Asp-212 and Lys-216 are shown for the resting state [blue, protein data bank (PDB) entry 6G7H] and partly isomerized state (cyan, PDB entry 6G7K). Maps are contoured at 4.0 root mean squared deviation. Cyan: positive difference density. Magenta: negative difference density. Reconstruction pump-probe delays from Eq. (11) are (a) 0.3 and (b) 760.7 fs.

We use the quasi-solution with $q=15001,j_{\text{max}}=20,\tilde{r}=1$, and *p* = 0, to calculate difference-electron-density maps. Intensities from the resting state frames are averaged, and maps are calculated following the procedure outlined in Ref. 10. Using *s* = 104 507 reconstructed frames, we compile a difference-electron-density movie that covers the first picosecond after photoactivation. Movies with various protein views are presented in the supplementary material. Maps show isomerization features compatible with the findings from binning-and-merging (Ref. 10), in particular a negative-positive pair of difference-density peaks corresponding to the movement of the C20 methyl group of the retinal chromophore (Fig. 22 and supplementary material Fig. 11). The observed negative features on the water cluster and the retinal polyene are also largely consistent with published data.^10^ However, especially features on the water cluster are controversially discussed.^21,22^ The negative-positive peak pairs around the Schiff base that indicate early events in the isomerization support the published structures but appear to be stronger and may allow a more detailed analysis of retinal isomerization under the conditions used.

**TABLE I.** Parameters used in the analyses reported in Fig. 9 and correlation coefficient between the retrieved dynamics and the ground truth.

$\sigma_{\text{jitter}}$	LPSA					NLSA							Binning	
	Parameters				CC	Parameters						CC	Parameters	CC
	*q*	$j_{\text{max}}$	*p*	$\tilde{r}$		*q*	*b*	${\text{log}}_{10}\;\epsilon$	*l*	*p*	$\tilde{r}$		Bin size	
0.1 $T_{\text{min}}$	4001	100	(q−1)/2	10	0.9826	101	3000	1.0	50	(q−1)/2	6	0.9854	1001	0.9424
0.3 $T_{\text{min}}$	4001	100	0	10	0.8903	4001	3000	1.0	50	(q−1)/2	10	0.8814	1201	0.8444
0.5 $T_{\text{min}}$	2001	100	0	4	0.7966	1001	3000	1.0	50	(q−1)/2	5	0.8006	2001	0.7827
1.0 $T_{\text{min}}$	4001	100	0	4	0.7434	1001	100	1.0	50	(q−1)/2	3	0.7442	4001	0.7275

## VI. DISCUSSION AND CONCLUSIONS

Because the extent of light-induced structural changes in the subpicosecond time range is limited, TR-SFX data from retinal proteins are expected to have a dominant static component, and a relatively small dynamic one. Using simulated data, we devise an improved method to handle missing observations, which is particularly relevant in the presence of a nonnegligible static component.

LPSA of the TR-SFX dataset from bR reveals that spurious frequencies contaminate low-order modes. Since these features are $j_{\text{max}}$-dependent, this prevents the solution from converging in the $j_{\text{max}}$ direction when contaminated modes are included in the reconstruction. In addition, the number of modes to be included cannot be derived from the singular-value spectra alone, since all modes have similar weight.

Such a spectral behavior as well as high-frequency contamination of the LPSA low-order modes is observed in our simulations when relatively high levels of Gaussian noise are included. In particular, by including a pixel-dependent static component (pedestal) and mimicking photon counting errors by considering that intensities are affected by Gaussian noise with $\sigma ={\left|J\right|}^{1/2}$, we observe a lack of singular-value decay (supplementary material Fig. 7) and chronogram contamination (Fig. 15 and supplementary material Fig. 8). As the size of the dynamical component of the signal decreases relative to noise, large, low-frequency spectral features from noise overwhelm the dynamical spectral features in the Fourier spectra of our signal. This hampers the accurate separation of dynamic components from noise.

Nevertheless, we observe that the first mode from the LPSA of bR TR-SFX intensity deviations is not significantly affected by high-frequency, physically implausible features. In addition, the measure of deviation from local linearity increases substantially after the first mode, and convergence with respect to both *q* and $j_{\text{max}}$ is assured when only one mode is used. We call this a *quasi*-solution since convergence with respect to the number of modes employed is not fulfilled. While this solution presents isomerization features compatible with published results, we emphasize that it cannot be assured that the trajectory of the system only explores one (concatenation space) dimension. The retrieval of other relevant modes may, in fact, be hampered by the high levels of noise.

For the synthetic data investigated in this study, our simulations clarify the limitations imposed by timing uncertainty on the accuracy of dynamics retrieval using the techniques considered. While with small values of timing uncertainty, LPSA and NLSA improve the quality of dynamics retrieval compared to binning-and-merging, a timing error $\sigma_{\text{jitter}}=1.0T_{\text{min}}$ hampers an accurate reconstruction (Fig. 9). While at high jitter values, most dynamical features are unresolved, we do not observe spurious features appearing in the solution with parameters optimized for numerical stability. We, nevertheless, observe high-frequency contamination of the chronograms at low values of *q* when the solution is not converged (supplementary material Fig. 4). We, therefore, stress that computational-parameter scans are of paramount importance. As discussed in Ref. 13, this task is less burdensome in LPSA compared to NLSA.

Good dynamics retrieval is obtained with $\sigma_{\text{jitter}}=0.5T_{\text{min}}$, which corresponds to a jitter FWHM of around $1.2T_{\text{min}}$. This means that features with periods of the order of a few tens of fs can be retrieved, if pump and probe pulse durations are sufficiently short, and a state-of-the-art timing-tool is used to timestamp the data.

In addition to pump-probe timing jitter, which is a stochastic phenomenon, quasi-systematic effects can also play a role. In particular, given the long duration of TR-SFX experiments (many hours or days), drifts in optical paths, due to unstable environmental conditions (pressure, temperature, and humidity), in the experimental hutch are expected. Depending on the details of the experimental setup, the optical paths pertaining to the diagnostic branch (incident on the timing-tool) and the pump branch (photoexciting the sample) of the pump laser can experience different variations in the environmental parameters. This can lead to a relative drift of one path compared to the other and give rise to a nonstochastic component of the timing uncertainty.

Compared to NLSA, LPSA is easier to understand and easier to use. LPSA and NLSA share the strategies of time-lagged embedding, time-domain subspace projection, and SVD. The difference is the specific way how vectors for the subspace-projection step are selected. LPSA does this based on a highly transparent, Fourier-based construction. NLSA, on the other hand, employs a diffusion-map algorithm. Apart from the less straightforward nature of such an algorithm, the high degree of incompleteness characterizing SFX data is incompatible with the assumption of dataset connectivity, which underlies diffusion maps and, therefore, requires large concatenation in NLSA. This reduces the advantage that NLSA may have for other problems. Furthermore, since in both NLSA and LPSA it is essential to verify that the final results are stable with respect to the computational parameters employed, the lower-dimensional the parameter space, the better. LPSA requires only one parameter ( $j_{\text{max}}$) for specifying the subspace-projection step, whereas NLSA requires three ( b,ϵ,l).

While timing errors seriously limit the accuracy of the retrievable dynamics, the major challenge we find in our dataset appears to reside in the large levels of noise at relatively low frequencies. While we cannot influence photon counting errors, which are intrinsic to the measurement and Poissonian in nature (and here modeled with Gaussians for simplicity), we can improve the sampling of the dynamics. In particular, we expect the low-frequency end of the noise spectrum to move to higher frequencies as the number of measured frames per unit time is increased, facilitating the spectral separation of dynamical features from noise.

## SUPPLEMENTARY MATERIAL

See the supplementary material for Figs. 1–11 and difference-electron density movies.

## Data Availability

The data that support the findings of this study are openly available in Zenodo at https://doi.org/10.5281/zenodo.7896581, Ref. 23, and in Github at https://github.com/CeciliaCasadei/dynamics-retrieval, Ref. 24.
